# Parkinson’s Disease Is Related to an Increased Risk of Ischemic Stroke—A Population-Based Propensity Score-Matched Follow-Up Study

**DOI:** 10.1371/journal.pone.0068314

**Published:** 2013-09-02

**Authors:** Ya-Ping Huang, Li-Sheng Chen, Ming-Fang Yen, Ching-Yuan Fann, Yueh-Hsia Chiu, Hsiu-Hsi Chen, Shin-Liang Pan

**Affiliations:** 1 Department of Physical Medicine and Rehabilitation, National Taiwan University Hospital Yun-Lin Branch, Yunlin, Taiwan; 2 School of Oral Hygiene, College of Oral Medicine, Taipei Medical University, Taipei, Taiwan; 3 Department of Nutrition and Health Sciences, Kainan University, Tao-yuan, Taiwan; 4 Department and Graduate Institute of Health Care Management, Chang Gung University, Tao-yuan, Taiwan; 5 Centre of Biostatistics Consultation, College of Public Health, National Taiwan University, Taipei, Taiwan; 6 Division of Biostatistics, Graduate Institute of Epidemiology, College of Public Health, National Taiwan University, Taipei, Taiwan; 7 Department of Physical Medicine and Rehabilitation, National Taiwan University Hospital, Taipei, Taiwan; 8 National Taiwan University College of Medicine, Taipei, Taiwan; The University of Hong Kong, Hong Kong

## Abstract

**Objective:**

The risk of stroke in patients with Parkinson’s disease (PD) remains controversial. The purpose of this population-based propensity score-matched longitudinal follow-up study was to determine whether there is an increased risk of ischemic stroke after PD.

**Methods:**

We used a logistic regression model that includes age, sex, pre-existing comorbidities and socioeconomic status as covariates to compute the propensity score. A total of 2204 patients with at least two ambulatory visits with the principal diagnosis of PD in 2001 was enrolled in the PD group. The non- PD group consisted of 2204, propensity score-matched subjects without PD. The ischemic stroke-free survival rates of the two groups were estimated using the Kaplan-Meier method. Stratified Cox proportional hazard regression with patients matched on propensity score was used to estimate the effect of PD on the occurrence of ischemic stroke.

**Results:**

During the three-year follow-up period, 328 subjects in the PD group and 156 subjects in the non-PD group developed ischemic stroke. The ischemic stroke-free survival rate of the PD group was significantly lower than that of the non-PD group (P<0.0001). The hazard ratio (HR) of stroke for the PD group was 2.37 (95% confidence interval [CI], 1.92 to 2.93, P<0.0001) compared to the non- PD group.

**Conclusions:**

This study shows a significantly increased risk of ischemic stroke in PD patients. Further studies are required to investigate the underlying mechanism.

## Introduction

Although Parkinson’s disease (PD) has been consistently reported to be associated with a higher risk of all-cause mortality in various epidemiologic studies [1]–[4], there are conflicting findings on the relationship between PD and stroke. Some studies have found that PD is linked to an increased risk of ischemic stroke and higher stroke-related mortality [5]–[7], with an estimated hazard ratio ranging from 1.5 to 3.6. Moreover, recent evidence has suggested that PD is associated with certain vascular risk factors, such as diabetes [8]–[11] and hypertension [12]. In contrast, other studies found that PD patients have a reduced frequency of vascular risk factors [13] and a lower risk of stroke [14], [15]. Because diabetes and hypertension are also risk factors for ischemic stroke, these vascular comorbidities may confound the association between PD and stroke. One way of minimizing the potential confounding effects of comorbidities is to match various clinical characteristics between subjects with and without PD [16], [17]. Propensity score matching methods are increasingly being used in observational studies to reduce bias [18], [19]. We therefore performed the current population-based, propensity score-matched longitudinal follow-up study to investigate whether there is an increased risk of ischemic stroke after the occurrence of PD.

## Materials and Methods

### Data Source

The data used in this study were obtained from the complete National Health Insurance (NHI) claim database in Taiwan for the period 2000 to 2003. The NHI program has been implemented in Taiwan since 1995, and the coverage rate was 96% of the whole population in 2000 and 97% at the end of 2003, i.e. more than 21.9 million persons. It should be noted that the rationale for using the NHI database after 2000 is that, from Jan 1st, 2000, according to the rules of the Bureau of NHI, the NHI claim data have been encoded using the standardized International Classification of Disease, 9^th^ Revision, Clinical Modification (ICD-9-CM).

### Ethics Statement

To keep individual information confidential to satisfy regulations on personal privacy in Taiwan, all personal identification numbers in the data were encrypted by converting the personal identification numbers into scrambled numbers before data processing. This study was exempt from full review by the National Taiwan University Hospital Research Ethics Committee and the need for informed consent was waived because the data used in this study consisted of de-identified secondary data released for research purposes and were analyzed anonymously, which complies with the regulations of the Department of Health, Executive Yuan, Republic of China.

### Study Subjects and Design

We used a prospective propensity score-matched cohort design to study the effect of PD on the risk of developing subsequent ischemic stroke. To control for potential confounding from imbalance in clinical characteristics, we used propensity score matching to create comparable cohorts between patients with and without PD [20], [21]. The study population consisted of a PD group and a non-PD group, both selected from Taiwanese residents in the complete NHI claim database in 2001, in which more than 21.6 million persons were registered.

The PD group consisted of subjects aged 40 years or older who had received a principal diagnosis of PD (ICD-9-CM code 332.0) in ambulatory medical care visits between January 1st, 2001 and December 31, 2001. To maximize case ascertainment, only patients who had at least 2 ambulatory visits with the principal diagnosis of PD and had been treated with anti-Parkinson medication (at least one of the following: amantadine, biperiden, bromocriptine, entacapone, levodopa, pergolide, pramipexole, ropinirole, selegiline, and trihexyphenidyl) in this period were initially considered for inclusion in the PD group (n = 17445). The index visit was defined as the first ambulatory visit during which a principal diagnosis of PD was made. The exclusion criteria for the PD group were: (1) a previous diagnosis of any type of PD (ICD-9-CM code 332.0, 332.1) during 2000 (n = 13554) to increase the likelihood of identifying only new incident PD cases in 2001; (2) a previous diagnosis of any type of stroke (ICD-9-CM codes 430–438) before the index ambulatory care visit (n = 6462); and (3) a previous diagnosis of other extrapyramidal disease, abnormal movement disorders, or cerebral degeneration (ICD-9-CM codes 333, 331) before the index ambulatory care visit (n = 2568), resulting in the exclusion of 15230 subjects because of one or more of these criteria. A total of 2215 subjects was identified in the PD group.

### Covariates and Propensity Score Matching

The information of pre-existing comorbidities, including diabetes (ICD-9-CM code 250), hypertension (ICD-9-CM codes 401–405), hyperlipidemia (ICD-9-CM code 272), coronary heart disease (ICD-9-CM codes 410–414 and 429.2), chronic rheumatic heart disease (ICD-9-CM codes 393–398), and other types of heart disease (ICD-9-CM codes 420–429), were acquired by tracking all the ambulatory medical care and inpatient records in the NHI database in the year before the index visit. The case ascertainment for these medical comorbidities was defined from 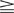
1 hospital discharge or 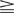
2 ambulatory visits with a relevant principal or secondary diagnosis code. Previous studies have suggested that the risk of stroke may be affected by socioeconomic status such as geographical regions, levels of urbanization, and income levels [22], [23]. Therefore, these factors are also taken into account as variables in assessing the risk of stroke. The information of the geographical location of residency of each subject was obtained from the population household registry. The geographical location of residency was classified into Northern, Central, Eastern, and Southern Taiwan. In accordance with Taiwan National Health Research Institute publications [24], urbanization levels in Taiwan are classified into 7 strata, with level 1 referring to the “most urbanized” and level 7 referring to the “least urbanized” communities. However, since there were relatively small number of subjects in levels 5, 6, and 7, these 3 levels were merged into a single group and labeled as level 5. For the income-level, we used the insured payroll-related amount as a proxy for income (0, NT$1 to NT$15840, NT$15841 to NT$25000, NT$25001; NT$ indicates new Taiwan dollar). Note that we selected NT$15840 as the first cutoff point of income level because this is the government-stipulated minimum wage for full-time employees in Taiwan. Since the household registry information is not available in 11 subjects out of the 2215 subjects in the PD group, these 11 subjects were excluded from the analysis. The final PD group consisted of 2204 subjects.

The non-PD group was taken from the remaining subjects without a diagnosis of PD in the same 2001 NHI claim database. We assigned the first ambulatory medical care visit during 2001 as the index ambulatory visit. The exclusion criteria for recruiting subjects into the non-PD group were: (1) a previous diagnosis of any type of PD (ICD-9-CM code 332.0, 332.1) before the index visit; (2) a previous diagnosis of any type of stroke (ICD-9-CM codes 430–438) before the index visit; and (3) a previous diagnosis of other extrapyramidal disease, abnormal movement disorders, or cerebral degeneration (ICD-9-CM codes 333, 331) before the index visit.

The information of pre-existing co-morbidities and socioeconomic status were acquired using the same methods described above. Because the number of subjects in the NHI database is very large, we used a two-stage method to select the propensity score-matched non-PD group [25]. For each subject in the PD group, we first randomly sampled 20 age and sex-matched non-PD subjects who met the abovementioned criteria. A total of 44080 non-PD subjects was initially sampled. In the second stage, a logistic regression model including age, sex, pre-existing co-morbidities and socioeconomic status as covariates was used to predict the probability (i.e. propensity score) of PD. An 8-to-1 greedy matching algorithm [20] was then used to identify a unique matched control from the 44080 non-PD subjects for each PD patient according to the propensity score. A total of 2204 subjects was selected in the propensity score-matched non-PD group.

### Outcome

All ambulatory medical care records and inpatients records for each subject in the propensity score-matched PD and non-PD groups were tracked from their index visit until the end of 2003 and mortality data for the subjects who died during the follow-up were obtained from the national mortality registry. The date of the first occurrence of a principal diagnosis of ischemic stroke (ICD-9-CM codes 433-437) within the follow-up period was defined as the primary endpoint. The case ascertainment for stroke required 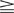
1 hospital discharge or 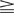
2 ambulatory medical care visits with the principal diagnosis of stroke. All subjects were followed from the index visit to the first occurrence of ischemic stroke, death, or end of follow-up.

### Statistical Analysis

The Chi-square test and student’s t test were used to examine differences in demographic variables, comorbid medical disorders and propensity scores between the PD and non-PD groups. The ischemic stroke-free survival curves of the propensity-score matched PD and non-PD groups were generated using the Kaplan-Meier method and the difference in survival between these two groups was assessed using the log-rank test. Stratified Cox proportional hazard regression with patients matched on propensity score was used to estimate the effect of PD on the occurrence of ischemic stroke. An alpha level of 0.05 was considered statistically significant for all analyses. The analyses were performed using SAS 9.2 software (SAS Institute, Cary, NC).

## Results

Table 1 shows the demographic and clinical characteristics of the PD and non-PD groups before propensity score matching. The PD group had a higher prevalence of certain pre-existing medical comorbidities, including diabetes (P<0.0001), hypertension (P<0.0001), coronary heart disease (P<0.0001), chronic rheumatic heart disease (P = 0.0432), and other heart disease (P<0.0001) than the non-PD group. There were also significant differences in the distribution of monthly income, urbanization level, and geographic region between the PD and non-PD groups. The PD groups had higher propensity score than the non-PD group (P<0.0001). After propensity score matching, the matched cohorts were well-balanced in terms of all observed covariates (Table 2). There was no statistically significant difference in all the baseline characteristics between the PD group and matched non-PD group (Table 2).

**Table 1:** Demographic characteristics and comorbid medical disorders for the Parkinson’s disease (PD) and non-PD groups before propensity score matching.

	PD group	Non-PD group	
Variables	(N = 2204)	(N = 44080)	*p* value
Sex (women)	1116 (50.6)	22320 (50.6)	1.0000
Age (years)	71.3±9.6	71.1±9.6	0.3563
Diabetes (yes)	393 (17.8)	6231 (14.1)	<0.0001
Hypertension (yes)	953 (43.2)	16367 (37.1)	<0.0001
Hyperlipidemia (yes)	210 (9.5)	3734 (8.5)	0.0828
Coronary heart disease (yes)	435 (19.7)	6460 (14.7)	<0.0001
Rheumatic heart disease (yes)	30 (1.4)	411 (0.9)	0.0432
Other heart disease (yes)	360 (16.3)	4962 (11.3)	<0.0001
Atrial fibrillation (yes)	32 (1.5)	511 (1.2)	0.2131
Monthly income			<0.0001
NT$0	871 (39.5)	15491 (35.1)	
NT$1–NT$15840	513 (23.3)	9588 (21.8)	
NT$15841–NT$25000	736 (33.4)	16752 (38.0)	
?NT$25001	84 (3.8)	2249 (5.1)	
Urbanization level			<0.0001
1 (most urbanized)	439 (19.9)	8335 (18.9)	
2	297 (13.5)	4621 (10.5)	
3	504 (22.9)	9820 (22.3)	
4	367 (16.6)	7386 (16.8)	
5	597 (27.1)	13918 (31.5)	
Geographic region			0.0114
Northern	937 (42.5)	19221 (43.6)	
Central	400 (18.2)	8036 (18.2)	
Southern	814 (36.9)	15278 (34.7)	
Eastern	53 (2.4)	1545 (3.5)	
Propensity score	0.051±0.014	0.048±0.012	<0.0001

Data are expressed as N (%) or mean ± SD.

US $1 =  NT $34 in 2001.

**Table 2:** Demographic characteristics and comorbid medical disorders for the Parkinson’s disease (PD) and non-PD groups after propensity score matching.

	PD group	Non-PD group	
Variables	(N = 2204)	(N = 2204)	*p* value
Sex (women)	1116 (50.6)	1090 (49.5)	0.4335
Age (years)	71.3±9.7	71.4±9.4	0.6772
Diabetes (yes)	393 (17.8)	381 (17.3)	0.6348
Hypertension (yes)	953 (43.2)	977 (44.3)	0.4662
Hyperlipidemia (yes)	210 (9.5)	221 (10.0)	0.5770
Coronary heart disease (yes)	435 (19.7)	426 (19.3)	0.7324
Rheumatic heart disease (yes)	30 (1.4)	27 (1.2)	0.6892
Other heart disease (yes)	360 (16.3)	351 (15.9)	0.7125
Atrial fibrillation (yes)	32 (1.4)	32 (1.4)	1.0000
Monthly income			0.7573
NT$0	871 (39.5)	878 (39.8)	
NT$1–NT$15840	513 (23.3)	532 (24.1)	
NT$15841–NT$25000	736 (33.4)	720 (32.7)	
?NT$25001	84 (3.8)	74 (3.4)	
Urbanization level			0.7849
1 (most urbanized)	439 (19.9)	442 (20.0)	
2	297 (13.5)	289 (13.1)	
3	504 (22.9)	497 (22.6)	
4	367 (16.6)	398 (18.1)	
5	597 (27.1)	578 (26.2)	
Geographic region			0.9824
Northern	937 (42.5)	947 (43.0)	
Central	400 (18.2)	403 (18.3)	
Southern	814 (36.9)	801 (36.3)	
Eastern	53 (2.4)	53 (2.4)	
Propensity score	0.051±0.014	0.051±0.014	0.9780

Data are expressed as N (%) or mean ± SD.

US $1 = NT $34 in 2001.

**Figure 1:**
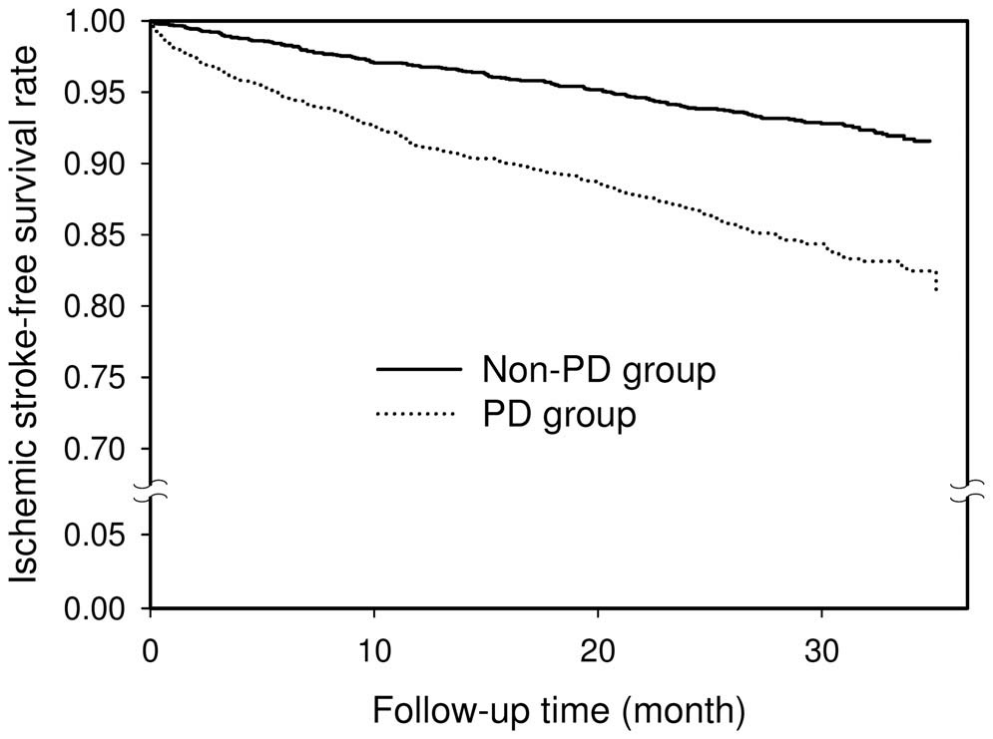
Three-year ischemic stroke-free survival rates for the propensity score-matched Parkinson’s disease (PD) group (dotted line) and the non-PD group (solid line).

The median follow-up time was 29.0 months (interquantile range 8.9 months). The number of stroke events and the hazard ratios (HR) of stroke for the two propensity score-matched groups are presented in Table 3. Of the 2204 patients with PD, 328 developed ischemic stroke during 4627.8 person-years of follow-up, giving an incidence rate of 70.9 (95% CI, 63.4 to 79.0) per 1000 person-years. Of the 2204 subjects in the non-PD group, 156 had an ischemic stroke during 5173.8 person-years of follow-up, giving an incidence rate of 30.2 (95% CI, 25.6 to 35.3) per 1000 person-years. The HR of stroke for the PD group was 2.37 (95% confidence interval [CI], 1.92 to 2.93, P<0.0001). The three-year ischemic stroke-free survival rates for the two groups are shown in Figure 1. The PD group had a significantly lower 3-year ischemic stroke-free survival than the non-PD group (P<0.0001).

**Table 3:** Number of ischemic stroke events and the hazard ratio of stroke for the matched Parkinson’s disease (PD) and non-PD groups.

	PD group	Non-PD group
Variables	(N = 2204)	(N = 2204)
Ischemic stroke events, N	328	156
Risk per 1000 person-yr (95% CI)	70.9 (63.4 to 79.0)	30.2 (25.6 to 35.3)
Hazard Ratio (95% CI)	2.37 (1.92 to 2.93)*	1.00

* P<0.0001.

## Discussion

The present population-based longitudinal follow-up study showed that newly diagnosed PD was associated with an increased risk of developing subsequent ischemic stroke. The three-year ischemic stroke-free survival rate for patients with PD was significantly lower than that for the non-PD group. We propose the following possible explanations for the positive association between PD and ischemic stroke.

First, although the etiology of PD is not fully understood, there is evidence that oxidative stress contributes to dopamine cell degeneration in PD [26]–[28]. Oxidative stress is considered to play an important role in endothelial dysfunction and the pathogenesis of atherosclerosis [29], [30], which may increase the risk of cardiovascular events [31]. We therefore hypothesize that the link between PD and ischemic stroke may be attributed to a common pathogenesis pathway, namely, oxidative stress, and the occurrence of PD may indicate higher cumulative oxidative stress, leading to a higher risk of ischemic stroke in the PD group.

Second, orthostatic hypotension (OH) has been recognized as one of the main non-motor symptoms of PD [32], [33]. One recent meta-analysis showed that the pooled estimate of the prevalence of OH was 30% in patients with PD [33]. OH has been suggested as a risk factor of ischemic stroke [34], [35]. Hence, we speculate that the PD related OH may also contribute to the higher risk of ischemic stroke in PD patients.

In the present study, we found that the PD group had a higher prevalence of diabetes and hypertension than the non-PD group before propensity matching (Table 1). In contrast, a case-control study reported that newly diagnosed PD patients have a reduced frequency of vascular risk factors, such as diabetes, hypertension, and dyslipidemia, compared to controls [13]. However, in this previous study, the controls were selected from patients admitted with other neurological diseases, rather than from the general population, which may result in unrepresentative findings. Moreover, recent evidence has shown that diabetes [8]–[11] and hypertension [12] are associated with an increased risk of developing PD, which is consistent with our finding that the PD group had a higher prevalence of diabetes and hypertension.

A strength of the present study is the use of a longitudinal population-based insurance database, which enabled us to identify all incident cases of ischemic stroke and establish a temporal relationship between PD and ischemic stroke. In addition, we used propensity score matching to minimize the potential confounding effects of all the included covariates. Most studies on the association between PD and stroke risk have used a cross-sectional or case-control study design [14], [36], [37] to evaluate prevalent stroke cases, rather than a longitudinal follow-up study design to identify incident cases. Consequently, little was known about the temporal relationship between occurrence of PD and subsequent development of stroke. Moreover, some studies were performed on selected clinical series of patients [13], [15] rather than by recruiting study subjects from the general population, which may result in unrepresentative findings. Since our study was a large population insurance-based cohort study and the temporal sequence between PD and stroke was ordered, i.e. PD preceded stroke, our findings provide evidence for a temporal association between PD and stroke. Such a temporal relationship is essential for establishing a causal connection.

This study is subject to several potential limitations. First, the diagnosis of PD, stroke, and medical comorbidities was determined by the ICD codes from the NHI claim database and there may be concern about the diagnostic accuracy of the database. However, the Bureau of NHI has formed different audit committees that makes it a rule to randomly sample the claim data from every hospital and reviews charts on a regular basis to verify the diagnostic validity and quality of care. Accordingly, the NHI claim database is recognized as an established research database and independent studies have demonstrated the validity of the data [38], [39]. Moreover, since PD is a clinical diagnosis, we adopted a case ascertainment algorithm that required at least two ambulatory medical care visits with a principal diagnosis of PD and the use of anti-Parkinson medication to improve the diagnostic accuracy. Second, the NHI database lacks some information regarding lifestyle factors, such as smoking, alcohol consumption, physical inactivity, and obesity, which may have affected the interpretation of our findings. Of these factors, smoking is a risk factor of stroke [40], but seems to be less prevalent in PD patients [3], [41], so an effect of smoking is unlikely to explain the increased stroke risk in the PD group seen in this study. Third, although we excluded patients with a previous diagnosis of any type of stroke for the PD group, it is still possible that the PD group might include patients who have had previous silent stroke or transient ischemic attack that have not been clinically diagnosed and therefore, would not be recorded in the NHI database. These undetected strokes may potentially contribute to both the development of PD and an increased risk of subsequent ischemic stroke. Fourth, chronic infectious burden and elevated inflammatory markers, such as C-reactive protein and interleukin-6, have been associated with the development of atherosclerosis and increased risk of stroke [42]–[44]. Nevertheless, data regarding inflammatory markers are lacking in the NHI database, it is therefore difficult to evaluate the potential effects of inflammatory markers on the association between PD and stroke. Further studies are required to investigate this specific issue. Finally, most of the inhabitants in Taiwan are of Chinese ethnicity and it is uncertain whether our findings can be generalized to other ethnic groups.

### Conclusions

This population-based, propensity score-matched longitudinal follow-up study shows an increased risk of ischemic stroke after diagnosis of PD. Further studies are needed to confirm this finding and investigate the underlying mechanism of this association between PD and ischemic stroke.
